# Beyond knowledge transfer: integrating critical evaluation-knowledge translation framework for decolonising global health

**DOI:** 10.7189/jogh.16.03006

**Published:** 2026-02-20

**Authors:** Jayoung Park, Blair Chang, Sebin Jung, Woong-Han Kim, Jongnam Hwang

**Affiliations:** 1 Program in Global Surgery and Implementation Science, JW LEE Centre for Global Medicine, Seoul National University College of Medicine, Seoul, Republic of Korea; 2 Seoul National University Medical Research Centre, Seoul, Republic of Korea; 3 University of Miami Miller School of Medicine, Miami, Florida, USA; 4 Department of Human Systems Medicine, Seoul National University College of Medicine, Seoul, Republic of Korea; 5 Department of Thoracic and Cardiovascular Surgery, Seoul National University Children’s Hospital, Seoul National University College of Medicine, Seoul, Republic of Korea; 6 Division of Social Welfare & Health Administration, Wonkwang University, Iksan, Republic of Korea

## Abstract

Capacity-building programmes for health professionals in low- and middle-income countries often transfer technical knowledge without sufficient attention to community ownership. This suggests knowledge translation alone is insufficient; it must be integrated with critical evaluation to systematically support community participation. Here, we proposes an integrated framework to align knowledge translation with community ownership and balanced power. The framework reframes capacity building programmes as the governance of knowledge, power, and ownership, and provides actionable guidance for more sustainable and locally led systems.

## CHALLENGING THE ‘GOLD STANDARD’: NEED OF POWER BALANCE

Capacity-building programmes for health professionals have become a central strategy in the global health field, especially in low- and middle-income countries (LMICs), where workforce shortages persist [1–3]. Despite their widespread adoption, many existing capacity-building initiatives remain structured around vertical donor-recipient relationships [4,5]. In these programmes, Western scientific knowledge is frequently positioned as the unquestioned correct answer, with so-called ‘gold standards’ transferred unidirectionally from the Global North to the Global South [6]. While such approaches may yield short-term gains, they often risk reinforcing dependency. External experts lead programme design, implementation, and standard-setting, and when they eventually exit, their departure can leave behind increased reliance on outside technical assistance rather than sustainable local capacity [7,8].

To address these challenges, knowledge translation (KT) has emerged as a key approach in LMIC health professional capacity-building programs to bridge the ‘know-do gap’ [9–11]. However, when KT is implemented without systematic analysis of community ownership, it risks reproducing the same hierarchical power dynamics that capacity-building efforts seek to overcome [12,13]. These limitations reflect critiques within the decolonising global health literature, which argue that many global health interventions continue to reproduce colonial power relations by privileging external knowledge, actors, and standards over local epistemologies and decision-making [14,15]. From this perspective, closing the know-do gap is insufficient unless attention is paid to who defines knowledge, whose expertise is legitimised, and who controls the terms of action.

Situated within the movement to decolonise global health, frameworks such as the critical evaluation (CE) proposed by Chi *et al*. provide interrogating power and legitimacy in KT interventions in LMICs. In this framework, Chi and colleagues emphasise community participation and process alongside outcomes, engaging with power balance in participatory decision-making [16]. Integrating such frameworks into KT can help shift capacity-building programmes away from one-way knowledge transfer, mitigating the vertical donor.recipient relationships and reducing the risk of increased local dependency on external actors.

In this viewpoint, we explain why CE and KT must be integrated together, drawing on conceptual synthesis and programmatic experience, and propose, an actionable framework to strengthen KT strategies in capacity-building programmes. Grounded in more than a decade of a Trauma and Orthopaedics Capacity-building Program in Vietnam, our observations reveal the limits of one-way knowledge transfer and the need to address power balance and community participation more systematically. In response, we propose a CE-KT framework that combines critical questions with actionable strategies to help programme designers and policymakers embed decolonising global health principles into practice.

## WHY INTEGRATION MATTERS

### KT’s blind spot

KT aims to close the know-do gap – the persistent disconnect between research evidence and its application in practice [17,18]. A key element to this process is collaboration and co-learning between researchers and communities, which recognise that knowledge must be tested, reshaped, and validated in local practice [19,20]. KT is often conceptualised through the knowledge-to-action (KTA) cycle, which comprises two components: the knowledge creation and the knowledge action cycle. The knowledge creation cycle begins with broad inquiry, proceeds to synthesis, and culminates in the development of tools that translate evidence into practice, such as clinical guidelines or decision aids [19]. The action cycle involves applying knowledge in real-world settings; it is inherently nonlinear and requires continual refinement in response to barriers, community feedback, and evolving needs [19].

The challenge of KT lies in navigating power relations among the diverse communities involved in research, including patients, caregivers, policymakers, and practitioners [21–23]. KT is often constructed on the assumption that evidence is neutral and that the translation of evidence into practice is an apolitical process [21]. This framing obscures the ways in which power shapes whose knowledge is recognised as legitimate and who ultimately controls its implementation [12]. Moreover, the term ‘knowledge user’ reinforces power imbalances inherent in research relationships by positioning researchers as the primary producers of knowledge while casting stakeholders and research partners as passive recipients [12]. Addressing these imbalances requires interrogating power within KT processes through actionable strategies and critical questions, such as: What knowledge is valued? Who defines the standards? And who holds decision-making authority?

### CE’s contribution

In this regard, the components of CE provide guidance by reshaping power relations through community participation. CE is a participatory framework that challenges conventional global health evaluation practices focused primarily on downstream effectiveness [16]. It emphasises whether programme outcomes and processes reflect community values, priorities, and needs, placing ethical principles such as ownership, self-determination, and inclusion at the centre of evaluation [24]. CE proposes a three-level evaluation framework: upstream, midstream, and downstream. Upstream asks whether the intervention originated from community-expressed needs or was externally imposed [25]. Midstream examines whether programme objectives are in line with community-defined needs or externally imposed metrics [26,27]. Downstream measures the outcomes of a programme based on its predefined goals. Without upstream legitimacy and midstream alignment, technically successful programs may still be irrelevant to local contexts [26].

### Why we need both? A retrospective analysis of the Trauma and Orthopaedics Capacity-building Program

The experience from a 12-year collaboration between Vietnam and Korea illustrates why integrating CE into KT is essential. The Trauma and Orthopaedics Capacity-building Program was initiated in 2013 to support the establishment of a new Trauma and Orthopaedics Centre in 2015 [28] and was shaped by the Korean team’s initial aim to convey what they regarded as the gold-standard trauma system.

At the beginning of the capacity-building programme, core components were developed in response to Vietnam’s expressed needs, rather than being structured around the trauma system model. The contrast between the Korean team’s initial trauma system model and the Vietnamese team’s workforce-based priorities emerged as a recurring pattern across multiple years of programme records. From the Korean perspective, an effective trauma system is typically organised around a trauma team leader and coordinated by trauma surgeons from general surgery, thoracic surgery, orthopaedics, and neurosurgery, reflecting a highly standardised model of acute trauma care. Vietnamese counterparts, however, did not simply accept this model. Instead, they raised a more fundamental question about whose priorities the proposed system reflected and emphasised their own clinical realities. They prioritised orthopaedic, plastic and reconstructive, and burn surgical specialist in response to workforce capacity and the immediate service needs of the newly established centre.

This divergence shifted the Korean team’s understanding of the work: the central question was not what the ‘right’ model looked like, but whose priorities it reflected and who had the authority to decide. Meaningful and sustainable KT requires first understanding who holds decision-making power, whose knowledge is treated as legitimate, and how change can realistically occur within local systems. In response, the programme shifted its approach. Rather than insisting on the adoption of an externally defined trauma system, the Korean team accepted Vietnam’s request to proceed gradually. The introduction of high-level invitational training (from 2016 onward) and joint frontline workshops was identified as a deliberate programmatic response to this power and priority alignment.

At the same time, this experience revealed a limitation. Although these relational and dialogical efforts were crucial, they lacked a systematic way to embed power, ownership, and decision authority into KT practice. This gap highlighted the need for an actionable framework that could make these dimensions explicit and operational, directly motivating the development of an integrated CE-KT framework.

## INTRODUCING AN ACTIONABLE FRAMEWORK: INTEGRATED CE-KT FRAMEWORK

**Figure 1:**
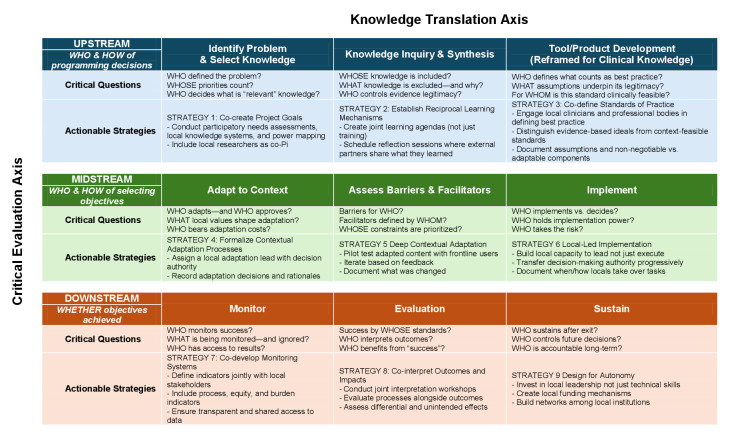
Detailed mapping of the CE-KT integrated framework.

Building on these insights, we propose an actionable integrated CE-KT framework to guide how LMIC capacity-building programmes can better align KT with community ownership and power balance (**Figure 1**). Building on the KTA cycle, the framework reframes each KT stage as a site of decision-making that can be critically examined across upstream, midstream, and downstream phases. Rather than treating problem definition, knowledge selection, and implementation as technical steps, the framework locates them within shifting configurations of authority, responsibility, and accountability. By embedding critical questions and actionable strategies at each CE-KT intersection, the framework enables evaluation of how capacity-building processes are co-produced in practice. Specifically, it allows examination of how training goals and clinical standards are negotiated, how adaptation decisions are made and by whom, and whether responsibility for implementation and sustainability is progressively transferred to local actors. In doing so, the framework moves beyond assessing whether knowledge was delivered or adopted and instead evaluates whether capacity building results in locally governed, contextually feasible, and durable practice.

The integrated CE-KT framework is designed to be scalable not through expansion of activities, but through selective institutionalisation of critical practices. The framework can be embedded within existing planning, training, and evaluation processes, such as routine programme reviews, clinical guideline adaptation committees, and monitoring and evaluation cycles. Its modular design allows implementers to prioritise specific stages or questions based on available resources, making partial adoption both feasible and meaningful. Over time, institutionalisation can occur through repeated use of shared decision-making procedures, documentation of adaptation rationales, and gradual normalisation of local authority in defining standards and priorities. The framework establishes a shared language that facilitates coordination, transparency, and accountability among multiple stakeholders involved in complex global health partnerships.

Redistributing decision-making authority is not possible through procedural tools alone; it may raise some challenges while applying the integrated CE-KT framework to capacity-building programmes. First, local stakeholders may not be able to articulate their needs at an early stage due to information asymmetries, not because they lack capacity, but because they lack exposure to alternative models. In other words, iterative need identification requires reciprocal learning rather than defining fixed priorities at the outset of a project. One way to address this challenge, as demonstrated in Vietnam’s Trauma and Orthopaedics Capacity-building Program, is to use invitational training for senior decision-makers and key clinical leaders to expand their exposure to alternative system designs and implementation options [29]. It is also a time-consuming process that requires long-term and sustained relationships, repeated interaction, and demonstrated accountability to achieve meaningful results. For this reason, ongoing monitoring and repeated data collection are needed to assess how authority, ownership, and practice evolve over time. Lastly, CE-KT’s capacity-building efforts remain vulnerable to brain drain, in that newly trained professionals may be drawn away from local systems, undermining long-term sustainability and local ownership [30].

## MOVING FORWARD WITH THE INTEGRATED CE-KT FRAMEWORK

Based on real-life experience, we propose an integrated CE-KT framework reframed not simply as the transfer of knowledge, but as the negotiation of decision-making authority, legitimacy of knowledge, and long-term community ownership. By embedding CE across the upstream, midstream, and downstream stages of the KTA cycle, the framework makes visible the often-understood power dynamics that shape why capacity-building efforts succeed or fail. By applying the framework, programme designers, implementers, and evaluators receive actionable guidance.

Methodologically, the CE-KT framework encourages evaluators to move beyond outcome-focused indicators toward process-sensitive and power-aware measures. It supports mixed-methods approaches that combine quantitative performance metrics with qualitative assessment of decision-making processes, adaptation pathways, and stakeholder roles. The framework also provides a structured basis for documenting when and how adaptations occur, enabling transparency and comparability without enforcing rigid fidelity to original models. As such, it offers methodological guidance for evaluating capacity-building initiatives in ways that are both contextually grounded and analytically rigorous.

The framework offers concrete entry points for improving the governance of capacity-building investments. Policymakers and funders can use it to require articulation of who defines priorities, who approves adaptations, and how responsibility is transferred over time. By emphasising shared authority, iterative review, and planning for autonomy from the beginning, the CE-KT framework aligns capacity-building policy with principles of local ownership, accountability, and sustainability. Importantly, it provides a common language that enables coordination across ministries, donors, implementing agencies, and frontline providers within complex health systems.
